# Development and implementation of an herbal and natural product elective in undergraduate medical education

**DOI:** 10.1186/1472-6882-12-57

**Published:** 2012-05-22

**Authors:** Kelly Karpa

**Affiliations:** 1 Department of Pharmacology, Pennsylvania State University College of Medicine, Mailcode R130, 500 University Drive, Hershey, PA, 17033, USA

**Keywords:** Complementary and alternative medicine, Dietary supplements, Herbal, Integrated therapy, Natural product, Undergraduate medical education

## Abstract

**Background:**

Medical students have consistently expressed interest in learning about alternative healing modalities, especially herbal and natural products. To fill this void in medical education at our institution, a novel elective was developed and implemented for fourth year medical students. This herbal/natural product course uses guest lecturers, classroom presentations, and active learning mechanisms that include experiential rotations, case-based learning, and team-based learning to increase student knowledge of herbal/natural product safety and efficacy.

**Methods:**

Knowledge outcomes were evaluated via administration of a pre- and post-course test (paired student *t*-test). End-of-course evaluations (Likert-type questions and narrative responses) were used to assess student opinion of knowledge and skills imparted by the elective and overall course content (mean, standard deviation).

**Results:**

Over three academic years, 23 students have enrolled in this elective. More than 60% of participants have been female and nearly half of the students (43%) have pursued residencies in primary care. Completion of the course significantly increased student knowledge of common herbal/natural product mechanisms, uses, adverse effects, and drug-interactions as determined by a pre- and post-course knowledge assessment (45% ± 10% versus 78% ± 6%; p < 0.0001). The course was highly rated by enrollees (overall course quality, 4.6 of 5.0 ± 0.48) who appreciated the variety of activities to which they were exposed and the open classroom discussions that resulted. While students tended to view some alternative medical systems with skepticism, they still believed it was valuable to learn what these modalities encompass.

**Conclusions:**

Development and implementation of a herbal/natural product elective that engages undergraduate medical students through active learning mechanisms and critical analysis of the literature has proven effective in increasing knowledge outcomes and is deemed to be a valuable curricular addition by student participants. In the future, it will be of interest to explore mechanisms for expanding the course to reach a larger number of students within the time, financial, and logistical constraints that currently exist.

## Background

Complementary and alternative medicine (CAM) disciplines are diverse medical and health care systems, practices, and products that are not part of conventional medicine. Three hundred distinct CAM modalities have been identified, including at least 20,000 herbal products that are available for consumer use [1]. In the United States, 38% of adults and 12% of children use one or more CAM modalities [2]. However, since health care providers often neglect to ask patients about CAM utilization, much CAM use may be medically unsupervised [3,4].

Lack of communication between physicians and patients about CAM can be dangerous, particularly with regard to patients’ use of herbal and natural products (H/NP). Due to dissatisfaction with allopathic health care - specifically concerns that traditional medications are too expensive, ineffective, or too focused on treating disease rather than maintaining good health - nearly one-fifth of Americans indicate concomitant use of H/NP and prescription medications [5]. This is concerning since H/NP are not pharmacologically inert, and potential exists for adverse events and drug-herbal interactions [5,6]. As a result of widespread H/NP use by patients, it is imperative that physicians be knowledgeable about common H/NP and be prepared to engage patients in conversations about dietary supplements.

Medical students indicate that they are not comfortable counseling patients about alternative healing modalities [7]. Yet, collectively, medical students consistently specify interest in learning more about these alternatives; this likely reflects students’ recognition of the social acceptability of this type of medicine [8]. Plant-based therapies, homeopathy, nutrition and naturopathy consistently rank among the top CAM therapies about which medical students would like to learn [7,9]. While over half of U.S. medical schools reportedly offer either an elective in CAM or CAM is included into required course work, tremendous diversity exists with the format of these courses, the content discussed, the amount of time dedicated to specific CAM topics, and the qualifications of the person(s) delivering the instruction [5,8,10-12].

In response to lack of guidelines or standardization among CAM curricula, the Institute of Medicine has recommended that health professional schools “incorporate sufficient information about CAM into the standard curriculum at the undergraduate, graduate, and postgraduate levels to enable licensed healthcare providers to competently advise their patients” [13]. At present, the majority of medical schools devote only two hours of time to H/NP in their curricula, and the way in which information is presented may lead students to believe that CAM modalities cannot be harmful [5,14]. However, students must understand that the “won’t hurt, might help,” mentality may not always apply to H/NP use. H/NP originate from plants or algae, and also include substances derived from natural sources like animal organs, marine exoskeletons, amino acids, or enzymes. Plant-based therapies, in particular, have potential to cause adverse effects and/or interfere with prescribed medications. Students may not be challenged to consider the potential harm that H/NP may cause, since few medical schools (8%) include evidence-based medicine or critical thinking into discussions of dietary supplements [5,15,16]. The lack of a critical approach to biologically-based CAM topics in undergraduate medical education is cause for concern.

Based upon the growing acceptance of H/NP by consumers and recurring student requests for more exposure to H/NP at our institution, a novel elective, “Herbal and Natural Products as Therapeutic Alternatives” (CAM 742) was developed in 2008 and implemented in 2009. Herein, the structure of the H/NP course is described. Furthermore, student views of H/NP after completing the course and outcome data that demonstrate improvement in student H/NP knowledge as a result of this new curricular component are discussed.

## Methods

### Course description

In response to student requests for coursework about herbal products, in 2007 the associate dean for clinical education at Pennsylvania State University College of Medicine (PSU COM) asked a pharmacology faculty member (who also happens to be a pharmacist) to develop an elective for fourth year medical students. The PSU COM is an academic medical center with approximately 580 medical students (1:1 male-to-female ratio) that is adjoined to a 484-bed teaching hospital. Additional clinical training takes place in 9 hospitals which are located throughout the south-central and eastern portions of the state of Pennsylvania. At the time the course was conceived and implemented, neither the PSU COM nor its affiliated hospital or clinic systems had formal H/NP infrastructure (no herbal formulary, no CAM clinics, no campus-wide resources specific for dietary supplements, etc.).

The “Herbal and Natural Products as Therapeutic Alternatives” (CAM 742) course was designed as a 4-week elective that places emphasis on literature appraisal and discussion. The elective is offered once during each academic year, and enrollment is limited to 9 students due to lack of CAM clinical sites in central Pennsylvania for student placement. In-class instructional time is comprised of two hours each day during the twenty day elective.

The goal of CAM 742 is to use an evidence-based medicine (EBM) approach to critically evaluate clinical trials that use H/NP as treatments for common medical conditions. Discussions of H/NP safety, efficacy, and purported mechanisms of action are explored. At the end of the course, students are able to: (1) Describe the current status of governmental regulations of H/NP; (2) compare and contrast quality, safety, and efficacy regulations between H/NP and drugs; (3) explain and describe the history of common alternative healthcare systems (e.g., Ayurveda, naturopathy, homeopathy, etc.); (4) Describe possible clinical applications, potential adverse effects, and current research regarding efficacy of common dietary supplements in comparison to prescription drugs for treatment of 10 common medical conditions; and (5) provide an appropriate response to patient inquiries about H/NP when given specific clinical scenarios.

To accomplish the course objectives, a multi-faceted program has been developed which includes: class-room lectures given by multidisciplinary faculty or guest lecturers, student presentations, as well as team-based learning (TBL) and case-based learning (CBL) clinical scenarios. Due to financial/travel/scheduling constraints of hosting the same guest experts each year, lectures have been electronically captured and a library of past guest lectures is available for student viewing. TBL and CBL instructional materials are based upon ten clinical case scenarios developed by the course instructor. During the course, students spend afternoons with community-based, licensed (MD, DO, RPh, NP, RN) or licensure-eligible (naturopathic physicians) providers or board-certified (clinical nutritionist) practitioners who routinely incorporate H/NPs and/or nutrition into therapeutic regimens for patients. Students observe these practitioners and participate in patient care to the extent that is appropriate for their knowledge and skill.

### Discussion and critical thought

Small-group discussions are a major component of each classroom session. Each day, students come to class prepared to share details of the patient case(s) they observed during clinical encounters the previous afternoon; students share the H/NP modalities/nutritional approaches the practitioner discussed with patients. Students are specifically asked to reflect upon new information gleaned from their clinical experiences and describe the methods by which they would have approached the clinical situation differently, based strictly upon their allopathic medical training. Furthermore, students share with the group any aspects of the CAM approaches they observed which they think may have merit or conversely be of concern, and students explain the modalities that they would like to explore further or incorporate into their clinical practices in the future. In addition to discussions of students’ clinical experiences, class-room based deliberations follow each faculty/guest lecture, as students’ perceptions of the information is explored on a deeper level. Furthermore, conversation and dialogue also follow each student presentation since a TBL exercise or CBL scenario has been authored by the course director around the topic of each student’s presentation.

### Classroom structure on student presentation days

Student presentations are based around common medical conditions (selected by students from a list provided by the faculty member) and three to five H/NP that patients may use to treat that condition, due to purported utility (Table 1). The faculty member (course director) does not endorse use of any of these products; instead, the focus of the course depends upon critical examination of literature and students form their own opinions based upon existing clinical trial data.

**Table 1:** Common Medical Conditions and Natural Products Discussed in Student Presentations and TBL/CBL Formats

**Medical Condition**	**Natural Products**
Alzheimer’s disease	Gingko biloba, huperzine A, vitamin E
Migraine	Feverfew, riboflavin, magnesium, butterbur, coenzyme Q10
Osteoarthritis	Glucosamine, chondroitin, S-adenyosylmethionine
Erectile dysfunction	L-arginine, DHEA, Panax ginseng
Benign prostatic hypertrophy	Saw palmetto, pygeum, stinging nettle
Menopause	Dong quai, black cohosh, soy
Hypercholesterolemia	Fish oils, oats, garlic, psyllium, policosanol
Heart disease	Vitamin E, coenzyme Q10, hawthorne, L-carnitine, L-arginine
Diabetes	Psyllium, chromium, cinnamon, American ginseng, gymnema, chromium, prickly pear, bitter melon
Depression	St. Johns wort, S-adenosylmethionine, inositol, fish oil

TBL, team based learning; CBL, case based learning.

In preparation for classroom presentations, students derive search terms and structure questions, assimilate evidence, and appraise the literature to form conclusions about H/NP safety and efficacy for their chosen topic (listed in Table 1). To accomplish these tasks, student presenters are specifically tasked with reviewing primary literature, creating a table of their findings, and interpreting/hypothesizing the reasons for discrepant outcomes observed across different studies. This encourages students to critically think about clinical trial designs (rather than relying solely upon someone else’s interpretation or biases that may be found in review articles) and draw their own conclusions regarding the available evidence base (or lack thereof). Non-presenting students are required to complete a peer-evaluation of the student presenter. Results of peer evaluations are compiled by the course director and returned to the student presenter, as a means of feedback that may be used to improve overall presentation skills prior to beginning residency.

To encourage non-presenting classmates to read about the topic in advance, a review article and/or a continuing medical education (CME) article from the Natural Medicines Comprehensive Database© (NMCD) is assigned. This assures that all students are prepared to contribute to active classroom discussions. In addition, consistent with TBL processes, a Readiness Assessment Test (RAT), comprised of three to five multiple choice questions (MCQ), is administered at the beginning of class. Students complete the RAT in teams (assigned by the faculty member), drawing upon each other’s knowledge from the assigned reading -- an approach that mimics a team-based approach to care.

Following each student presentation, a TBL or CBL clinical case (developed by the faculty member, based upon cases found in Shapiro, 2006 [17]) is distributed to each team of students. In TBL, a clinical scenario is presented, along with MCQ; however, there is not always a single best answer for these MCQs. Instead, each team of students must discuss the options, arrive at a group consensus, and be prepared to defend the teams’ choice to their peers. In CBL group discussions, each team develops a treatment plan for the clinical case and presents their treatment plan to the class. Treatment plans often involve: additional patient education and lifestyle counseling; discouraging patient use of specific H/NP due to pre-existing conditions, lack of efficacy data, or drug interactions for H/NP; encouraging use of H/NP based upon safety or efficacy data; or providing nutritional suggestions to the patients. These discussions become spirited as students are asked to vote for the treatment plan they would choose to follow for the patient (but they cannot select their own).

To facilitate discussions, students are given access to the NMCD, a web-based resource that provides evidence-based information about dietary supplements. This tool is particularly useful in identifying drug-interactions and side effects associated with H/NP, problems which may not be readily apparent from only reading the primary literature which is often limited by small numbers of participants. Students are also provided with information about other H/NP resources that may be of use (e.g., Consumer Labs, United States Pharmacopeia, etc.). During undergraduate medical education training, students have already become proficient with searching PubMed and many students incorporate information gleaned from the Cochrane database into their presentations.

### Evaluation methods

A 20-question pre-course and a post-course instrument that assesses H/NP knowledge is administered to all students. This instrument assesses students’ knowledge of dietary supplement regulatory issues, definitions pertaining to H/NP, potential for adverse events, putative mechanism(s) of action, drug-herbal interactions and contraindications, as well as clinical applications for various H/NP. The H/NP knowledge instrument is developed by the course instructor each year, based upon the topics that are scheduled to be covered in class. Some questions have been modified from Shapiro [17]. Answers to the pre-test are not reviewed with the students. Neither pre- nor post-course test results are incorporated into students’ grades; however, the instrument is used to assess changes in H/NP knowledge that occur during the four-week elective.

Final course grades are determined on the basis of in-class presentations; attendance, participation, and professionalism in class; team-answers submitted on RATs; professionalism/participation at afternoon sessions with clinical practitioners; and grade earned on a final course examination. The final course examination consists of multiple choice, short-answer, and essay-questions that challenge students to solve clinical scenarios. Course grades are not submitted until students complete all course evaluations, including evaluations of the half-day sessions with clinical practitioners, evaluations of each lecturer, and an end-of-course evaluation. The end of course evaluation consists of several questions which ask students to appraise (5 point scale; 1 = Less Satisfactory, 5 = More Satisfactory) the course in terms of overall quality, relevance to medical practice, course design, and encouragement of critical thinking. Students are also asked to rank their level of agreement (5-point scale; Disagree = 1, Agree =5) with statements pertaining to skills learned as a result of the course, as well as the perceived value of different course components. Finally, students are asked to respond to several narrative questions in which they describe their thoughts of the course in free form. Because this analysis utilizes existing data that is consistent with that acquired in educational settings, this work was deemed to be exempt from oversight by the Milton S. Hershey Medical Center Investigational Review Board (#37608EM).

### Data analysis

Cumulative data from the first three years that the course has been offered are included in this analysis. To quantitatively evaluate the impact of the CAM 742 course on students’ pre- and post-course knowledge assessments, a paired student *t*-test was used (GraphPad 6.0) with statistical significance set at p < 0.05. End-of-course student evaluations were examined descriptively through means and standard deviations, and a qualitative assessment of student comments regarding the course and themes that emerged are described.

## Results

### Demographics

Basic demographic information for students enrolled in the H/NP elective is depicted in Table 2. More females than males participated in the course each year that it has been offered. Nearly half (43%) of the students chose to enter a primary care residency following graduation. Other residency programs selected by students include: psychiatry (n = 2), emergency medicine (n = 2), ophthalmology (n = 2), anesthesiology (n = 2), pathology (n = 1), general surgery (n = 1), radiology (n = 1), research (n = 1), and orthopedics (n = 1).

**Table 2:** Demographics and Knowledge Outcomes

Year	Male N (%)	Primary Care Residency^a^ N (%)	Mean (±SD) Pre-Course Knowledge Assessment (%)	Mean (±SD) Post-Course Knowledge Assessment (%)	P value Knowledge Assessment
2009	2 (40)	2 (40)	54 (± 11)	83 (± 3)	0.004
2010	4 (44)	5 (55)	42 (± 11)	77 (± 6)	<0.0001
2011	3 (33)	3 (33)	42 (± 4)	76 (± 6)	<0.0001
Total	9 (39)	10 (43)	45 (± 10)	78 (± 6)	<0.0001

^*^Primary care defined as: Family Medicine, Internal Medicine, Pediatrics, Obstetrics/gynecology.

### Knowledge outcomes

Despite a small number of enrolled students, the mean scores on post-tests (example from 2011 in Additional file 1) increased significantly from pre-test scores (Table 2) each time the course was offered. Therefore, the format used in the 4 week elective is effective for increasing student knowledge of common H/NP.

### Student attitudes and opinions

The course has been consistently rated highly by students in all areas of course content assessed, including overall quality of the course, relevance of topics covered to the practice of medicine, encouragement of critical thinking, and overall course design (rated >4 on a 5 point scale) (Table 3). Students agree that the course provides them with relevant definitions, theory, and history underlying a variety of H/NP practices. They also indicate that the course improves their knowledge of clinical efficacy (or lack thereof) for a variety of H/NP. Furthermore, students also affirm that the course affords a better understanding of potential adverse effects associated with H/NP and provides an understanding of the current research supporting or refuting H/NP use. Importantly, following the course, students indicate that they know where and how to find reputable resources for evidence-based H/NP information. This knowledge provides students with confidence that they can effectively recommend or dissuade patients from using common H/NP, in specific clinical situations. Students also indicate that the NMCD is a valuable part of the course. Students appreciate the realization that they do not need to *know* all the answers since tools such as the NMCD can provide access to “just-in-time” learning and can be accessed at the point-of-care.

**Table 3:** Student Evaluation of Course

	**2009** Mean (±SD)	**2010** Mean (±SD)	**2011** Mean (±SD)	**Cumulative Years** Mean (±SD)
Course Provided **Definitions, Theory, History** of Alternative Health Systems	4.8 (0.45)	4.7 (0.71)	4.9 (0.33)	4.8 (0.48)
As a result of this course, I know **Clinical Efficacy** of H/NP	4.6 (0.89)	4.6 (0.73)	5 (0)	4.7 (0.59)
As a result of this course, I know **Adverse Events** associated with common H/NP	4.4 (0.55)	4.4 (1.01)	4.7 (0.50)	4.5 (0.70)
As a result of this course, I know the status of current H/NP **Research**	4.4 (0.55)	4.4 (0.73)	4.8 (0.44)	4.6 (0.58)
As a result of this course, I can find**EBM** H/NP Information	4.6 (0.89)	4.9 (0.33)	4.8 (0.44)	4.8 (0.48)
As a result of this course, I am able to confidently **Recommend or Dissuade** use of H/NP, given a particular patient situation	4.0 (0)	4.4 (0.73)	4.6 (0.52)	4.4 (0.57)
**Guest Lecturers** Were Valuable	4.6 (0.89)	4.6 (0.88)	3.9 (0.92)	4.3 (0.89)
**Experiential Afternoons** Were Valuable	4.2 (0.84)	4.2 (0.67)	3.9 (1.17)	4.1 (0.83)
**NMCD** Was Valuable	4.6 (0.89)	4.9 (0.33)	4.7 (0.71)	4.7 (0.58)
Course was **Relevant** to Practice of Medicine	NA	4.4 (0.53)	4.8 (0.44)	4.6 (0.50)
Course **Encouraged Critical Thinking**	NA	4.4 (0.53)	4.9 (0.33)	4.7 (0.49)
Rating of Overall **Course Design**	NA	4.7 (0.50)	4.7 (0.50)	4.7 (0.49)
Rating of Overall **Course Quality**	NA	4.7 (0.71)	4.6 (0.53)	4.6 (0.61)

NA, not asked; NMCD, Natural Medicine Comprehensive Database; H/NP, herbal and natural products; EBM, evidence-based medicine

Mean (±SD) student responses are based upon a 5 point scale. Scale anchors for questions pertaining to overall course quality, relevance to medical practice, course design, and encouragement of critical thinking are: 1 = Less Satisfactory, 5 = More Satisfactory. Scale anchors for questions pertaining to student level of agreement with statements relating to skills learned from the course and value of course components are: 1 = Disagree, 5 = Agree.

### Emergent themes

In addition to rating agreement with specific statements about the course on a Likert-type scale, students also provide narrative comments about their experiences in the course. These narrative comments provide additional insight into views about the course and H/NP in general.

Several themes have emerged when students comment about their learning experiences in the course. In all three academic years that the course has been offered, students have spoken highly of the “variety of activities” in which they could engage in this course, including the “opportunity to think through cases and evaluate the literature critically”. Along the same lines, it was mentioned that the “breadth of the course offered a great overview” of H/NPs. Furthermore, it was stated that “the informative background information, allowed for discussions and explorations of personal opinions and experiences”. Consistent with this were comments indicating that the course “increased insight into other ideas/practices; even if I did not agree with them; it was useful to broaden horizons”. Similarly, during the last two years that the course was offered, a theme emerged regarding the importance of a professor who “allows for a great deal of freedom, which serves to enhance our educational experiences” in this type of course offering. Moreover, another student commented, “Open conversations are necessary to make the classroom experience valuable; [it] requires a relaxed setting where students are not overly stressed about grades that will show up on their transcript come interview day.” When these comments are taken altogether, it appears that students place great value on both the broad variety of activities and information that is presented in the course, as well as the ability to learn the information in a relaxed and causal setting, with freedom to express dissenting opinions.

Over the years, guest lecturers participating in the course have had varied backgrounds. Guests have included: medical doctors, nurse practitioners, pharmacists, naturopathic physicians, nutritionists, practitioners certified in traditional Chinese medicine, Ayurvedic physicians, and research scientists. The wide range of topics presented by these providers is listed in Table 4. Overall, students have found exposure to alternative health systems beneficial (Table 4), although they have critically questioned some modalities. For example, students frequently indicate on their evaluations that “more concrete data” is needed following the presentations given by specific guest lecturers. Interestingly, this theme has surfaced for both MD- as well as non-MD providers, emphasizing the importance that medical students place on EBM prior to legitimizing any treatment or modality*, regardless of the training* of the individual espousing it.

**Table 4:** Specialty Lectures by Faculty or Guest Lecturers and Perceived Value

**Topic**	**Perceived Value per “This Topic Should be Offered Annually” Selected on Course Evaluation(N responding) %**
Vitamin D Supplementation	(17 of 18) 94%
Natural Methods to Prevent Depression	(9 of 9) 100%
Homeopathy	(17 of 20) 85%
Naturopathy	(8 of 8) 100%
Nutrition	(7 of 7) 100%
Probiotics	(16 of 16) 100%
Bioidentical Hormone Replacement Therapy	(14 of 14) 100%
Ayurvedic Medicine	(8 of 9) 89%
CAM Fellowship/Functional Medicine Board Certification Opportunities for Physicians	(not assessed)
Vitamins as Therapeutics	(5 of 5) 100%
Alternatives to Asthma, Allergies, Autism, and ADHD	(5 of 5) 100%
Natural Product Regulations in the United States	(not assessed)
10 Dietary Supplements Every Physician Should Know	(5 of 5) 100%
Omega-3 Fatty Acid Supplementation	(5 of 5) 100%
Traditional Chinese Medicine	(5 of 5) 100%

Generally, during classroom discussions, students tend to be most skeptical and critical of homeopathy and naturopathy, medical systems with which medical students are typically unfamiliar. In the case of homeopathy, students have difficulty conceptualizing how infinitesimally small dilutions of a substance can exhibit any pharmacologic activity, since homeopathy counters everything they have learned about pharmacologic dose–response relationships. On the other hand, from observation of classroom discussions, it appears the difficulty students have with naturopathy lies not with the *concept*, as much as the *practice*s they have observed from the providers during afternoon clinical experiences. Students are offended that these providers refer to themselves as “physicians”; medical students view their own training as “superior” to that of the naturopathic providers and find it insulting and misleading that another group of professionals refers to themselves as *physicians*. Furthermore, students have expressed concern regarding the medical information they have observed some naturopaths giving to patients. This purportedly has included belittling comments made regarding medical doctors’ knowledge about nutrition and health and wellness, as well as admonitions given to patients about the therapeutic modalities used by these providers (e.g. “Do not tell your doctor about this”).

In contrast to student skepticism about homeopathy and naturopathy, students in each class have consistently indicated that nutrition is an area that “we can all relate to” and have stated that “we need more [of this] stuff that will be useful to MDs.” Students uniformly indicate that the sessions spent with a nutritionist are valuable. In addition, students have also repeatedly cited probiotics as an area “we can all relate to and understand its usefulness in medicine”; another student explicitly stated, “I could see myself recommending this [probiotics] with my patients”.

When asked to comment about aspects of the course that students found most meaningful or useful, four individuals specifically cited that the NMCD will be an important resource for them in their future practice. Students also indicated that the student presentations/classroom discussions were particularly useful; they specifically referenced such things as “learning about H/NP that are commonly used by consumers”, “learning about efficacy and side effect profiles” for H/NP, the “discussions/critical thinking” that were evoked, and “developing treatment plans for cases you might actually encounter in practice” as valuable. One student commented, “In preparing my own presentation, trying to get solid data on CAM therapies was eye opening.” Several students found the experiential afternoons to be especially meaningful, and one stated, it was”important to see why patients choose natural products over medications, and learn about complaints patients have with primary care providers such that they seek out natural medicine. Learning that we do not devote enough time, explain things in detail, or listen to [patients] as well as they expect has been meaningful because I would like to incorporate those changes in my future practice. [It was] valuable to see who was seeking care and why.”

Students also commented on the skills and/or knowledge with which they felt more confident following completion of the H/NP course. Students mentioned that the course increased their confidence in counseling patients about H/NP, as they were now more knowledgeable about H/NP and their side effects, as well as more familiar with resources where evidence-based information about H/NP could be found. One student responded, “I'm less afraid to ask about CAM because I feel I have some knowledge to impart and know where to look for good information.” Another stated, “I am more open to discussing these products with patients now that I have a basis of knowledge and a better understanding of where to find legitimate and reliable information/facts about them.” Concurrently, after stating that his/her confidence in discussing H/NP with patients had increased as a result of the course, the same student also acknowledged, “I also feel more confident *dismissing* some forms of alternative medicine as ineffective.” Additionally, several students commented favorably about having an improved understanding of different CAM modalities. One individual stated that after graduation, “I may look into some of the alternative practitioners in the area where I practice, to get a sense of what they do. Are they quacks or can I utilize them as an adjunct to my practice to refer patients?”

## Discussion

### Impact of CAM 742 elective

Students have consistently given high ratings to the CAM 742 H/NP course. Certainly positive feedback is desirable, yet the true measure of a successful course is the extent to which outcomes – in this case knowledge -- improve. Based upon the pre- and post- course knowledge assessments, this four week course, comprised of clinical, didactic, and team-based learning, significantly improves student knowledge pertaining to H/NP. In 2010, Nicolao et al. referenced the scarcity of objective knowledge assessments in undergraduate medical education CAM coursework [18]. Thus, the objective results achieved herein, through use of a pre- and post-course knowledge assessment, are the first to demonstrate improved knowledge outcomes from a CAM course in the undergraduate medical education arena.

As an elective rather than a required course, CAM 742 is similar to most CAM courses offered at other medical schools [5]. Like other CAM courses, our H/NP course makes use of instructors and guests to provide lectures, group discussions, case studies, and office visits to CAM practitioners [5,7,9]. However, the H/NP course we offer is different from those at other medical schools in several notable ways. First, the extensive classroom contact time (>40 hours) is more than double that offered in most undergraduate medical CAM courses. Second, our H/NP course is offered by a basic science department rather than a clinical department, which likely allows for a heightened level of critical analysis of supplements from a mechanistic and side effect point of view [5]. Finally, in other CAM courses that have been described in the literature, students are exposed to numerous integrated therapeutic modalities including: acupuncture; mind-body interventions like relaxation, imagery, prayer, biofeedback, hypnosis, or yoga; manipulative methods like chiropractic and massage; as well as energy therapies like therapeutic touch. However, no courses that focus exclusively on H/NP were identified in reviews of undergraduate medical education literature.

The intention in developing CAM 742 was not to train physicians to be competent to practice exclusively using H/NP in lieu of conventional pharmacotherapies, but to educate physicians who can effectively *communicate* about these products and advise patients regarding safe and effective uses of these alternative supplements. This course is intended to assist students in forming their own opinions regarding the role of H/NP in clinical medicine through critical analysis of the medical literature and assimilation of evidence for H/NP efficacy and safety. Additionally, it is anticipated that students enrolled in the course will become knowledgeable with common drug-herbal interactions, will exhibit a heightened awareness of open dialogue between patients and their physicians, and will have an increased understanding of different medical systems and modalities espoused by alternative healthcare providers.

Although dietary supplements are regulated as *foods* (rather than drugs) in the United States, future physicians must be cognizant that patients frequently use dietary supplements in search of pharmacologic outcomes, rather than to correct a dietary deficiency. As a result of participation in CAM 742, it is envisioned that students will be better able to communicate to patients that dietary supplements cannot be ignored as pharmacologically inert, but rather must be treated as *medications*, complete with the potential to cause troublesome adverse effects or clinically-relevant drug interactions.

Each year the course has been offered, the therapeutic relationship between patients and healers (loosely defined as either physicians or alternative medicine practitioners) and its patient-centeredness (who seeks treatment from alternative providers and why), along with positive perspectives on the role of good nutrition in healthy living, have become key components of classroom discussions. By inviting non-MD, non-PhD guest lecturers to talk to the medical students (nurse practitioners, naturopathic physicians, physician’s assistants), we have incorporated multiprofessional education in our medical school. It is hoped that these multi-professional educational experiences will facilitate inter-professional communication, increase mutual respect, enhance understanding of the roles played by different professionals, and ultimately lead to increased interdisciplinary collaborations, research, and improved patient care.

### Incorporating CAM into medical education

According to the literature, most medical students are in favor of integrating CAM into the medical system [19]. While most medical students relay positive personal experiences with CAM, the majority of students also recognize the need for additional research efforts in this area [18,20]. Similar to what we have observed in CAM 742, students at other medical schools have also raised skepticism about certain healing practices, including homeopathy [18]. Generally, first year medical students tend to view CAM more favorably than senior level students, perhaps reflecting the curricular emphasis on EBM and randomized, clinical trials during training [21].

In the United States, a major obstacle for integrating different healthcare approaches into medical education is the existence of separate educational tracks for CAM training versus allopathic medical education. Before conventional healthcare providers and providers from CAM disciplines could ever be educated and care for patients in a collaborative fashion—with the mutual goal of improving patient outcomes -- the mistrust and suspicion that has fostered this separation would need to be resolved.

Many physicians are entirely resistant to their patients using any dietary supplements; as a result, H/NP are not typically integrated within undergraduate medical coursework or seen as a value within training institutions [22]. Consistent with this, H/NP are often viewed as something to be learned “in addition” to medical training (e.g., electives), rather than as a valid body of knowledge that should be *integrated* into the curriculum at all levels. A contrasting view, however, is apparent within the Cuban medical system where herbal therapies are integrated into required medical school course work. In Cuban pharmacology courses, for example, students learn the properties, actions, contraindications, and interactions for the 49 medicinal herbs that the Cuban medical authority has approved as therapeutics [13].

Given the large number of patients in the United States who use H/NP therapies, it would seem warranted to propose that the basic pharmacologic principles/properties for a core list of commonly used H/NP should be taught to U.S. health professions students as well. Such an action would likely foster discussions between patients and healthcare providers about H/NP efficacy and safety. Yet, to evoke change in the *status quo*, a cooperative team effort would be required on the part of educators who acknowledge that this material is worth teaching to students simply because *patients want to know*. According to Brokaw et al., medical students should be trained in the fundamentals of dietary supplements so they can elicit relevant information from patients, assess potential risks, and guide treatment accordingly [5]. Furthermore, to best accomplish this, according to Brokaw, students must receive training in critical scientific evaluation of the literature through enlistment of faculty in basic science departments, who can impart a critical perspective [5]. It is believed that the CAM 742 course has accomplished this directive.

### Limitations

Admittedly, there are limitations to the present analysis. First, the CAM 742 course is offered as an elective at a single institution, which may limit the generalizability of our experiences. At our institution, the H/NP course is offered once each year and enrollment is rather limited. The course is offered on a restricted basis due to (a) the large time commitment required of the course director and the (b) logistics involved with the running the course. During the month that this course is offered, the course director dedicates 40 classroom contact hours to the endeavor, in addition to preparing out-of-class for case discussions, grading assignments, and coordinating experiential afternoon rotations with CAM practitioners, etc. Furthermore, the number of students that can be accommodated in the CAM 742 elective at any one time is limited by the availability of local practitioners in central Pennsylvania who are willing to host our students for the experiential afternoon portion of the course. This may be more or less of a problem in various locations around the country; presumably large metropolitan areas would find this to be less of an impediment. Lack of credentialed providers available to participate in medical school courses has been previously identified as a limiting factor for other CAM courses, and, in the absence of adequate funding, clearly limits the course topics that can be presented by guest lecturers [23]. Due to the time-intensive nature and logistical challenges, a course such as we have implemented may not be feasible at all institutions.

With the limited offering of the CAM 742 course each year, scheduling challenges result for students who truly desire to take the class; not all students who wish to take the elective are able to participate. Moreover, students who elect to enroll in the H/NP course may be more favorably predisposed to H/NP than the general population of students. No attempts have been made to quantify students’ personal experiences with dietary supplements before enrolling in the class. However, it has been apparent in classroom discussions that some enrollees enter the course with more favorable views of CAM *a priori* compared to other students. Along those lines, we have had more female than male enrollees each time the course has been offered; this may be consistent with previous studies in which females have been shown to have a greater interest in CAM approaches and are more inclined to report favorable uses of CAM compared to their male counterparts [23,24].

Another limitation stems from the inability to directly measure the impact that this course has on student-patient communications in clinical encounters. Each year, the H/NP course is offered near the end of the 4^th^ year. For many students, the H/NP elective is the last class they take prior to graduation. Therefore, the extent to which this course improves patient interactions and facilitates discussions with patients regarding use of H/NP in clinical practice is impossible to ascertain. If a mechanism existed by which we could determine the impact of the H/NP course on actual practice in the clinics, this would be the ultimate method by which the success of the course could be measured. It is conceivable that, in the future, a series of targeted questions sent to alumni or residency directors could be developed to assess this.

Our experiences developing and implementing an H/NP course are relevant to other health professional educational programs. This type of curriculum is not only pertinent to medical students, but is also applicable to other allied health professional students such as pharmacy, physician assistant, and graduate level nursing students. Nonetheless, sustainability of such a curriculum requires three key components: a dedicated and enthusiastic faculty member, student access to evidence-based dietary supplement resources, and availability of multi-professional practitioners who are willing and able to provide lectures and host students at their practice sites.

## Conclusions

In conclusion, patients deserve physicians who are sensitive to societal desires for use of H/NP and who are knowledgeable about both the potential value and limitations of such treatments. This elective for undergraduate medical students attempts to fill the void. The strengths of this curriculum include: (1) a focus on primary literature and critical thinking which are imperative for decision-making and enable students to use existing evidence to distinguish between useful or useless interventions; (2) clear thematic content presented in the context of case-based and team-based learning mechanisms that feature common medical conditions and real-life problems/questions faced by patients; (3) experiential components that enable students to observe practical applications of alternative medical systems; (4) communications with alternative healthcare clinicians to facilitate student understanding of patient co-management across various healthcare modalities; and a (5) focus on the importance of *talking to patients* and asking about alternative therapies they may be using. Overall, the content areas covered in this course have been deemed to be relevant to the practice of medicine by students enrolled in the course, the course design has been highly rated by students, and significant improvement in student knowledge of H/NP has resulted.

## Abbreviations

CAM = Complementary and alternative medicine; CBL = Case-based learning; EBM = Evidence based medicine; H/NP = Herbal and natural products; NMCD = Natural medicine comprehensive database; TBL = Team-based learning.

## Competing interests

The author declares no competing interests.

## Authors’ contributions

K.K. developed the CAM 742 course, is the course director, and authored this manuscript.

## Authors’ information

K.K. is a pharmacist and pharmacologist who serves as the director of medical pharmacology instruction at Penn State University College of Medicine.

## Pre-publication history

The pre-publication history for this paper can be accessed here:

http://www.biomedcentral.com/1472-6882/12/57/prepub

## Supplementary Material

Additional file 1 2011 CAM 742 Knowledge Assessment. Click here for file

## References

[B1] Hoellein A Griffith GH Lineberry MJ A complementary and alternative medicine workshop using standardized patients improves knowledge and clinical skills of medical students Altern Ther Health Med 2009 15 30 34 19943574

[B2] Barnes PM Bloom B Nahin R CDC National Health Statistics Report #12 2007 Complementary and Alternative Medicine Use Among Adults and Children, United States December 10, 2008 19361005

[B3] Giveon SM Liberman N Klang S Kahan E A survey of primary care physicians’ perceptions of their patients’ use of complementary medicine Compl Ther Med 2003 11 254 260 10.1016/S0965-2299(03)00114-6 15022662

[B4] Winslow LC Shapiro H Physicians want education about complementary and alternative medicine to enhance communication with their patients Arch Intern Med 2002 162 1176 1181 10.1001/archinte.162.10.1176 12020190

[B5] Brokaw JJ Tunnicliff G Beat RU Saxon D The teaching of complementary and alternative medicine in U.S. Medical Schools: A Survey of Course Directors Acad Med 2002 77 876 881 10.1097/00001888-200209000-00013 12228082

[B6] Norred CL Zamudio S Palmer SK Use of complementary and alternative medicines by surgical patients J Am Assoc Nurse Anesth 2000 68 13 18 10876447

[B7] Hoellein AR Lineberry MJ Kifer E A needs assessment of complementary and alternative medicine education at the University of Kentucky College of Medicine Med Teach 2008 30 e77 e81 10.1080/01421590801938860 18484445

[B8] Greenfield S Innes M Allan T Wearn A First year medical stduents’ perceptions and use of complementary and alternative medicine Complementary Ther in Med 2002 10 27 32 10.1054/ctim.2002.0501 12442820

[B9] Nicolao M Tauber MG Heusser P How should complementary and alternative medicine be taught to medical students in Switzerland? A survey of medical experts and students Med Teach 2010 32 50 55 10.3109/01421590902825123 20095775

[B10] Wetzel MS Eisenberg DM Kaptchuk TJ Courses involving complementary and alternative medicine at US medical schools JAMA 1998 280 784 787 10.1001/jama.280.9.784 9729989

[B11] Institute of Medicine Committee on the Use of Complementary and Alternative Medicine Complementary and Alternative Medicine in the United States National Academy of Sciences, Washington, DC January 2005

[B12] Frenkel M Fry A Heliker D Finkle T Yzaquine D Bulik R Sierpina V Lessons learned from complementary and integrative medication curriculum change in a medical school Med Educ 2007 41 205 213 10.1111/j.1365-2929.2006.02654.x 17269955

[B13] Appelbaum D Kligler B Barrett B Frenkel M Guerrera MP Kondwami KA Lee BB Tattelman E Natural and traditional medicine in Cuba: Lessons for U.S. Medical education Natural and traditional medicine in Cuba: Lessons for U.S. Medical education. MEDICC Review 2008 10 43 48 21483356

[B14] Yildirim Y Parlar S Eyigor S Sertoz OO Eyigor C Fadiloglu C Uyar M An analysis of nursing and medical students’ attitudes towards and knowledge of complementary and alternative medicine (CAM) J Clin Nurs 2010 19 1157 1166 10.1111/j.1365-2702.2009.03188.x 20492061

[B15] Kemper KJ Amata-Kynvi AA Sanghavi D Whelan JS Dvorkin L Woolf A Samuels RC Hibberd P Randomized trial of an internet curriculum on herbs and other dietary supplements for health care professionals Acad Med 2002 77 882 889 10.1097/00001888-200209000-00014 12228083

[B16] Sampson W The need for educational reform in teaching about alternative therapies Acad Med 2001 76 248 250 10.1097/00001888-200103000-00011 11242574

[B17] Shapiro K Natural Products: A Case-Based Approach for Health Care Professionals 2006 American Pharmacists Association, Washington, D.C

[B18] Nicolao M Tauber MG Marian F Heusser P Complementary medicine courses in Swiss medical schools: actual status and students’ experiences Swiss Med Wkly 2010 140 44 51 20131118 10.4414/smw.2010.12760

[B19] Tiralongo E Wallis M Integrating complementary and alternative medicine education into the pharmacy curriculum Am J Pharm Edu 2008 72 74 10.5688/aj720474 PMC2576413 19002274

[B20] Brinkhaus B Joos S Willich SN Hahn EG Complementary and alternative medicine in German medical schools Med Teach 2005 27 180 10.1080/01421590500047021 16019344

[B21] DeSylvia D Stuber M Fung CC Bazargan-Hejaz S Cooper S The knowledge, attitudes and usage of complementary and alternative medication of medical students Evid Based Complement Altern Med 2011 Article ID 985243 10.1093/ecam/nen075 PMC3137293 19073778

[B22] Corbin WL Shapiro H Physicians want education about complementary and alternative medicine to enhance communication with their patients Arch Intern Med 2002 62 1176 1181 10.1001/archinte.162.10.1176 12020190

[B23] Chaterji R Tractenberg RE Amri H Lumpkin M Amorosi ASBW Haramati A A large-sample survey of first- and second- year medical student attitudes toward complementary and alternative medicine in the curriculum and in practice Altern Ther 2007 13 30 35 10.1089/act.2006.13103 PMC4371739 17283739

[B24] Boehmer S Karpa K Evaluating the value of a web-based natural medicine clinical decision tool at an academic medical center BMC Health Serv Res 2011 11 279 10.1186/1472-6963-11-279 22011398 PMC3206841

