# Supplementary material for: Development and implementation of an herbal and natural product elective in undergraduate medical education
Source: BMC Complement Altern Med. 2012 May 22;12:57. doi: 10.1186/1472-6882-12-57 (PMC3358235; doi:10.1186/1472-6882-12-57)
Supplement: Additional file 1 — 2011 CAM 742 Knowledge Assessment. [file 1472-6882-12-57-S1.doc]

2011 CAM 742 Knowledge Assessment

1. A mixture of lipid-lowering dietary supplement products claims to "support healthy cholesterol levels" on the bottle. This labeling is:

a. Allowed by the FDA as a structure/function claim

b. Not allowed by the FDA since these products cannot make claims for specific medical indications

2. If a product that claims to "improve airway function" is proven to be unsafe, who is responsible for removing the product from the market?

A. Federal Trade Commission

B. Food and Drug Administration

C. Manufacturer

D. National Center for Complementary and Alternative Medicine

E. United States Pharmacopeia

3. A patient wishes to give a natural product to her mother who has been diagnosed with Alzheimer's dementia. Her daughter does not want to give her any more prescription drugs, because she believes they are all toxic and states that her mom is taking too many already. Her mother takes clopidogrel, amlodipine, simvastatin, and aspirin. If the daughter insists upon using a supplement, the product that is LEAST LIKELY to increase bleeding in this patient is:

A. Gingko biloba

B. Huperzine A

C. Vitamin E

4. A patient is currently using donepezil. It is reasonable to recommend huperzine A simultaneously because the mechanisms of the drug and supplement might complement each other.

a. True

b. False

5. A patient wishes to try a natural product for migraine prophylaxis. She has diarrhea-predominant, irritable bowel syndrome and liver disease (Hepatitis B and C positive) from years of IV drug abuse. She reports ragweed allergies. Which of the following products would be LEAST likely to aggravate any of her pre-existing conditions?

A. Magnesium

B. Feverfew

C. Riboflavin

D. Butterbur

6. Which of the following is TRUE concerning coenzyme Q10?

A. Causes intolerable gastrointestinal side effects at standard dosages

B. Endogenous levels rise with aging

C. Is a necessary for mitochondrial energy production

D. It may increase blood glucose levels.

7. Dietary supplement products that contain which of the following have a high risk of being of poor product quality and are expensive?

A. Feverfew

B. S-adenosylmethionine

C. Chondroitin

D. Glucosamine

8. Which of the following natural products is most likely to increase the risk of bleeding?

A. Chondroitin

B. Fish oils

C. Glucosamine

D. S-adenosylmethionine

9. Select the correct mechanism of L-arginine in increasing penile blood flow.

A. L-arginine is converted to nitric oxide, which relaxes the vasculature and causes vasodilation

B. L-arginine is converted to prostacyclin which relaxes the vasculature and causes vasodilation

C. L-arginine causes general CNS relaxation which results in improved erection

10. A patient with diabetes would like to try something natural for managing erectile dysfunction. He has an insulin pump, but is poorly controlled. He has been having "sugar lows". Due to his hypoglycemic episodes, you counsel him that he should probably AVOID:

A. DHEA

B. Panax ginseng

C. Yohimbine

11. Saw palmetto has a mechanism of action similar to finasteride.

A. True

B. False

12. Stinging nettle has strong evidence supporting its role in symptom reduction in men with benign prostatic hyperplasia

A. True

B. False

13. Which of the following is TRUE concerning black cohosh?

A. If beneficial for hot flushes, relief will be apparent almost immediately (within days)

B. There are many recognized drug interactions because it inhibits hepatic CYP450s

C. The risk of the supplement contributing to a breast cancer diagnosis is considered to be small

D. There have been case reports of pulmonary toxicity

14. A woman wishes to try bioidentical hormone replacement therapy (BHRT) for osteoporosis prevention. She has already had a stroke and her mother and sister had breast cancer. A trial period of BHRT in this patient is :

A. Safer than using prescription hormone replacement therapy

B. Not advised

15. A patient wishes to use St. John's wort. The clinician should recommend the following nonprescription product to the patient, too:

A. Aspirin, taken 1 hour before

B. Psyllium, daily for constipation

C. Magnesium, daily for constipation

D. Sunscreen, use as needed

16. Which of the following may cause hypertensive crisis and should be avoided in patients with bipolar disorder due to risk of precipitating mania?

A. Fish oils

B. Inositol

C. S-adenosylmethionine

D. St John's Wort

17. Although soy-containing foods are probably okay in this patient population, soy supplements are probably best avoided in patients with a history of:

A. Breast cancer

B. Eczema

C. High cholesterol

D. Hypertension

18. Which of the following is UNLIKELY to increase the risk of bleeding?

A. Fish oils

B. Garlic

C. Policosanol

D. Psyllium

19. Which of the following supplements has an “FDA-authorized qualified health claim” for use in type 2 diabetes to reduce insulin resistance?

A. American ginseng

B. Cassia cinnamon

C. Chromium picolinate

D. Prickly pear cactus

20. The Daily Recommended Intake (DRI) of which vitamin or mineral was recently re-evaluated because many believe that the DRI is too low for optimal health?

A. Magnesium

B. Selenium

C. Lycopene

D. Vitamin D

E. Beta-carotene
